# Perioperative outcomes following tranexamic acid use in arthroscopic rotator cuff repair

**DOI:** 10.1016/j.jseint.2026.101769

**Published:** 2026-07-27

**Authors:** Jeffrey Coombs, Nicholas Morriss, Kelsey Loyd, Christopher Dussik, Andrew Jae-Min Park, Dylan N. Greif, Sandeep Mannava

**Affiliations:** Department of Orthopaedic Surgery, University of Rochester, University of Rochester School of Medicine, Rochester, NY, USA

**Keywords:** Tranexamic acid, Post-operative opioids, Arthroscopy, Rotator cuff repair, Healthcare utilization, Post-operative outcomes

## Abstract

**Background:**

Tranexamic acid (TXA) is widely used in orthopedic surgery to mitigate intraoperative and post-operative bleeding. Current literature supports its use in arthroscopic rotator cuff repair (RCR) to increase visualization, decrease operative time, and improve immediate post-operative pain. This study aimed to assess post-operative outcomes of arthroscopic RCR with and without perioperative TXA. We hypothesize that TXA use will result in decreased post-operative complications, including less pain and therefore fewer opioid prescriptions.

**Methods:**

The TriNetX database was queried to assess post-operative outcomes after arthroscopic RCR with and without perioperative TXA performed between January 1, 2010, and December 31, 2024. Post-operative outcomes were evaluated at one month and followed for one year. Evaluated outcomes included emergency department visits, hospital admissions, superficial and deep surgical site infections, pulmonary embolism, and number of opioid prescriptions. One-to-one greedy propensity score matching was performed to control for potential confounding variables, and odds ratios were calculated to assess the overall risk of the outcomes of interest between cohorts. A *t*-test was performed to evaluate differences in the number of opioid prescriptions. Statistical analysis was set at *P* < .05 for all analyses.

**Results:**

We identified 120,236 RCR without perioperative TXA and 7,645 RCR with perioperative TXA. After matching based on demographics and medical comorbidities, 7,645 patients remained in each cohort. Analysis at both 1 month and 1 year post-operative demonstrated that patients receiving perioperative TXA were associated with fewer opioid prescriptions (27.9% versus 31.2% at 30 days and 43.5% versus 47.2% at 1 year, *P* < .0001). The perioperative TXA cohort also had decreased odds of readmission at 1 year but not 30 days (0.2% versus 0.6%, *P* < .0001). No significant differences were found in pulmonary embolism rates or superficial and deep surgical site infections.

**Conclusion:**

This is the first large-scale, database study demonstrating perioperative TXA in the setting of arthroscopic RCR was associated with significantly improved post-operative outcomes, particularly fewer post-operative opioid prescriptions at 30 days and 1 year and a decrease in hospital admissions at 1-year follow-up. This updates the limited current literature surrounding TXA use during shoulder arthroscopy, with some studies suggesting improved visual field and decreased immediate post-operative pain, while others positing no clinical difference in pain reduction. Future well-designed prospective studies are needed to elucidate these associations more fully.

Tranexamic acid (TXA) has become an important adjunct in orthopedic surgery due to its proven ability to reduce perioperative blood loss and decrease transfusion requirements. TXA was first adopted in arthroplasty, where it was found to consistently decrease the need for allogeneic transfusion without increasing the risk of thromboembolic complications.^8^^,^^18^ The use of TXA has expanded rapidly into orthopedic sports procedures, particularly arthroscopic shoulder surgery. Recent randomized controlled trials and meta-analyses demonstrate that both intravenous and intra-articular TXA can improve intraoperative visualization, reduce blood loss, shorten operative time, and decrease early post-operative pain following arthroscopic rotator cuff repair (RCR)^3^,^5^^,^^7^,^10^^,^^11^^,^^21^. Systematic reviews further demonstrate TXA is a safe adjunct to decrease intraoperative bleeding without increased risk of thromboembolic events or major complications.^9^^,^^19^ However, little research has been done evaluating the impact of TXA on post-operative pain after discharge.

Effective pain management is critical for improving patient satisfaction, accelerating recovery, and reducing opioid use. Adjuncts that can decrease opioid usage aid early recovery and minimize side effects.^12^^,^^20^ These in turn are associated with decreased healthcare utilization and cost of care.^1^ As a result, optimizing pain control not only enhances outcomes but also plays a key role in reducing the overall cost of care in arthroscopic surgery.^6^^,^^17^

The present study aims to evaluate the impact of perioperative TXA on outcomes such as emergency department visits, readmission, surgical site infections (SSIs), and the number of opioid prescriptions following arthroscopic RCR. We hypothesize that perioperative TXA will reduce the number of post-operative opioid prescriptions without increasing adverse post-operative outcomes or healthcare utilization.

## Methods

This is a retrospective analysis of data derived from TriNetX, a multinational, multi-institution research network, which collects data from 106 distinct healthcare organizations and from over 100 million patients. TriNetX aggregates data derived from individual medical records, including demographics, medications, laboratory test values, International Classification of Diseases (ICD) diagnoses, and Current Procedural Terminology (CPT) procedural codes. Information from the database is aggregated and de-identified using a standard defined in Section §164.514(a) of the HIPAA (Health Insurance Portability and Accountability Act) Privacy Rule. The process by which the data are de-identified is attested to through a formal determination by a qualified expert as defined in Section §164.514(b) (1) of the HIPAA Privacy Rule. The TriNetX database was queried on April 8, 2026. As information reported in the database is aggregated and de-identified, it was exempt from institutional review board review. There was no funding required for this study. The REporting of studies Conducted using Observational Routinely-collected health care Data (RECORD) guidelines were reviewed and implemented in manuscript preparation.^4^

We evaluated patients who underwent arthroscopic RCR from January 1, 2010, to December 31, 2024, who either received or did not receive TXA within 1 day, before or after their surgery date, and were between 18 and 90 years of age. Arthroscopic RCR was identified with CPT code 29827. TXA administration was identified by RxNorm code 10691.

Demographic characteristics and comorbidities were identified using CPT and ICD-10th Revision (ICD-10) codes, as well as algorithmically converted ICD-Ninth Revision codes. Demographic information obtained for the study included age and sex. Comorbidities assessed included hypertension (I10), obesity (E65-E68), nicotine dependence (F17), diabetes (E08-E13), ischemic cardiac disease (I20-I25), kidney disease (N17-N19), chronic obstructive pulmonary disease (J44), epilepsy (G40), opioid abuse (F11.1), and opioid dependence (F11.2). Outcomes were followed from one day to one year post-operatively with analysis performed at 30 days and 1 year and included number of opioid prescriptions, hospital readmission, emergency department visits, superficial SSI, deep SSI requiring a return to the operating room, sepsis, deep vein thrombosis (DVT), pulmonary embolism (PE), acute kidney injury, sepsis, and mortality. Systematized Nomenclature of Medicine-Clinical Terms, ICD-10, and CPT codes used during our post-operative outcome analysis are included in Supplemental Table 1.

Chi-squared tests were utilized to assess significance for categorical variables, and independent-sample *t*-tests were used for continuous variables. Logistic regression incorporating age at the time of surgery, demographic variables, and medical comorbidities was used to generate propensity scores. One-to-one greedy matching without replacement was then performed to create matched cohorts for comparative analysis. Odds ratios and 95% confidence intervals were used to determine significance between cohorts. Statistical significance was set at *P* < .05 for all analyses.

## Results

### Demographics and comorbidities

A total of 127,881 arthroscopic RCRs were identified between January 1, 2010, and December 31, 2024. Among these, 7,645 patients received perioperative TXA compared to 120,236 who did not receive TXA. After matching, 7,645 patients remained in each cohort. Matching eliminated all differences in demographic variables and comorbidities (Table I).

**Table I:** Baseline characteristics of TXA and no TXA groups before and after propensity score matching.

Variable	Before PSM				After PSM			
	TXA (n = 7,645)	No TXA (n = 120,236)	Std diff	*P* value	TXA (n = 7,645)	No TXA (n = 7,645)	Std diff	*P* value
Total N	7,645	120,236	—	—	7,645	7,645	—	—
Age at index	58.7 ± 10.2	58.2 ± 10.3	0.052	<.001	58.7 ± 10.2	58.8 ± 10.2	0.009	.593
Female	3,261 (42.7%)	51,822 (43.1%)	0.009	.446	3,261 (42.7%)	3,244 (42.4%)	0.004	.781
Male	4,379 (57.3%)	68,235 (56.8%)	0.011	.366	4,379 (57.3%)	4,394 (57.5%)	0.004	.806
Diabetes mellitus	1,594 (20.9%)	20,754 (17.3%)	0.091	<.001	1,594 (20.9%)	1,583 (20.7%)	0.004	.826
Obesity	2,221 (29.1%)	26,357 (21.9%)	0.164	<.001	2,221 (29.1%)	2,230 (29.2%)	0.003	.873
Hypertension	3,758 (49.2%)	50,242 (41.8%)	0.148	<.001	3,758 (49.2%)	3,784 (49.5%)	0.007	.674
Ischemic heart disease	979 (12.8%)	13,785 (11.5%)	0.041	<.001	979 (12.8%)	963 (12.6%)	0.006	.698
Kidney disease	644 (8.4%)	7,172 (6.0%)	0.095	<.001	644 (8.4%)	615 (8.0%)	0.014	.394
COPD	426 (5.6%)	6,212 (5.2%)	0.018	.121	426 (5.6%)	395 (5.2%)	0.018	.266
Nicotine dependence	1,243 (16.3%)	15,401 (12.8%)	0.098	<.001	1,243 (16.3%)	1,222 (16.0%)	0.007	.644
Epilepsy	104 (1.4%)	1,456 (1.2%)	0.013	.249	104 (1.4%)	82 (1.1%)	0.026	.105
Opioid-related disorders	162 (2.1%)	1,774 (1.5%)	0.048	<.001	162 (2.1%)	134 (1.8%)	0.027	.100

*Std diff*, standardized difference; *PSM*, propensity score matching; *TXA*, tranexamic acid; *COPD*, chronic obstructive pulmonary disease.

Continuous variables are presented as mean ± SD; categorical variables as n (%).

Demographics and comorbidities after matching of all patients undergoing arthroscopic rotator cuff repair with and without perioperative TXA. After matching all standardized mean differences were <0.10, indicating appropriate covariate matching.

### Matched perioperative outcome analysis

Matched analysis was performed between patients undergoing arthroscopic RCR with and without TXA administration. Prior to matching, multiple significant differences existed in the incidence of comorbidities between the 2 cohorts, including opioid disorders. However, after matching, no significant differences remained (Table I). In the year following surgery, the patient cohort with TXA had significantly fewer total opioid prescriptions (27.9% versus 31.2% at 30 days and 43.5% versus 47.2% at one year, *P* < .0001). Patients with TXA also had significantly lower rates of readmission at 1 year (odds ratio = 0.31; 95% confidence interval 0.17-0.57) but no significant differences in readmission at 30 days. There were no significant differences in coded rates of DVT or PE between the 2 cohorts. There was no significant difference between superficial or deep SSI (Table II).

**Table II:** Outcomes after rotator cuff repair with vs. without TXA.

Outcome	30 days		1 yr	
	TXA (n = 7,645)	No TXA (n = 7,645)	TXA (n = 7,645)	No TXA (n = 7,645)
ED visits	**261** (3.4%)	**294** (3.8%)	**1,287** (16.8%)	**1,362** (17.8%)
	OR 0.884 (0.746–1.047)		OR 0.934 (0.859–1.015)	
	**P = .154**		**P = .109**	
Superficial SSI	**25** (0.3%)	**32** (0.4%)	**135** (1.8%)	**147** (1.9%)
	OR 0.781 (0.462–1.318)		OR 0.917 (0.724–1.161)	
	**P = .353**		**P = .471**	
Pulmonary embolism	**11** (0.1%)	**21** (0.3%)	**43** (0.6%)	**52** (0.7%)
	OR 0.523 (0.252–1.086)		OR 0.826 (0.551–1.239)	
	**P = .077**		**P = .354**	
Acute renal failure	**24** (0.3%)	**27** (0.4%)	**238** (3.1%)	**243** (3.2%)
	OR 0.889 (0.512–1.541)		OR 0.979 (0.816–1.174)	
	**P = .674**		**P = .817**	
Opioid prescriptions	**2,131** (27.9%)	**2,382** (31.2%)	**3,324** (43.5%)	**3,610** (47.2%)
	OR 0.854 (0.797–0.915)		OR 0.860 (0.807–0.916)	
	**P < .001**		**P < .001**	
Readmission	—	—	**14** (0.2%)	**45** (0.6%)
	—		OR 0.310 (0.170–0.565)	
	—		**P < .001**	
DVT	—	—	**30** (0.4%)	**48** (0.6%)
	—		OR 0.624 (0.395–0.985)	
	—		**P = .041**	
Sepsis	—	—	**31** (0.4%)	**48** (0.6%)
	—		OR 0.644 (0.410–1.013)	
	—		**P = .055**	
Deep infection	—	—	**203** (2.7%)	**185** (2.4%)
	—		OR 1.100 (0.899–1.346)	
	—		**P = .355**	

*OR*, odds ratio, reported with (95% confidence interval); *SSI*, surgical site infection; *DVT*, deep vein thrombosis; *TXA*, tranexamic acid; *ED*, emergency department.

Bold *P* values indicate statistical significance (*P* < .05).—Suppressed: fewer than 10 events observed at this time point.

Matched 30-day and 1-year perioperative complications and opioid prescriptions for patients undergoing arthroscopic rotator cuff repair with or without perioperative TXA. Values in bold are statistically significant.

The columns with “–” represent suppressed values as there were fewer than 10 observed events at that given time point.

## Discussion

We found that perioperative administration of TXA around arthroscopic RCR was associated with a decrease in the number of post-operative opioid prescriptions at 30 days and 1 year and readmission at one year without increasing rates of coded venous thromboembolism. This is the largest known study to evaluate a decrease in the number of post-operative opioid prescriptions at the population level within arthroscopic surgery following TXA administration.

Over 250,000 arthroscopic RCRs are performed annually in the United States, and the rate is expected to increase in coming years.^13^ Rehabilitation following RCR is a critical component of improving outcomes but can be slowed by poor post-operative pain control and remains an area of clinical concern.^15^ Optimization of post-operative outcomes is multifactorial and dependent on factors such as patient selection, nutrition, operative technique, and rehabilitation protocol.^15^ The use of intra-articular or intravenous TXA is one adjunct of interest to improve pain control and potentially, therefore, improve post-operative outcomes after arthroscopic RCR.

The use of TXA in arthroscopic shoulder surgery has been shown to improve intraoperative visualization, reduce blood loss, shorten operative time, and decrease early post-operative pain following arthroscopic RCR.^5^^,^^10^^,^^11^^,^^21^ This study demonstrated a potential additional benefit to TXA administration during arthroscopic RCR by decreasing the number of post-operative opioid prescriptions, as well as the risk of hospital readmission at one year. However, readmission data should be interpreted cautiously, as we are unable to attribute this to shoulder-specific causes. No difference in rates of DVT, PE, or SSI was found. Similarly, Mahatme et al^16^ demonstrated an association with improved pain control in the acute and chronic phases after RCR using the TriNetX database. Our study adds to this knowledge by corroborating the results with a larger sample size and also demonstrating an association between TXA use and decreased healthcare utilization at one year follow-up.

Early evidence in shoulder arthroscopy corroborates the ability of TXA to decrease post-operative pain.^2^^,^^5^^,^^14^ Liu et al^14^ found that intravenous TXA prior to incision improved visualization, subjective pain control, and decreased opioid requirements in the acute post-operative period, while finding no change on operative time, length of stay, or estimated blood loss. In a separate randomized control trial, Bildik et al^5^ found that intra-articular TXA decreased acute post-operative pain scores, total operative time, and improved visual clarity within shoulder arthroscopy. Both studies followed the cases of single surgeons and consistently demonstrated improved pain control in the acute post-operative phase regardless of route of administration. The present study adds to these results by demonstrating an association with decreased post-operative opioid prescriptions, even outside of the acute post-operative period, at the population level. In our study opioid prescriptions are used as a proxy for analgesic requirement, and while it suggests pain may be better controlled with TXA use, due to limitations of the TriNetX database and its lack of patient-reported outcomes, we are unable to definitively say patients had improved pain control. In addition, this study showed an association with decreased healthcare utilization with lower risk of inpatient admission at one year, but not at 30 days.

This study has limitations. The results depend on the accuracy and completeness of procedural and billing code documentation which may be unreliable. For example, matched comorbidities such as opioid-related disorders may be more or less prevalent than coded and may represent a confounding variable. Furthermore, as a database this study is unable to evaluate granular details such as imaging findings, operative details, route and duration of TXA administration, quantity of opioids within each prescription, or the morphine equivalents prescribed. Therefore, while we did find a statistical difference in number of opioid prescriptions, the clinical significance is unclear. The findings of the number of opioid prescriptions and hospital admissions are purely associative and may be impacted by a mediating variable we were unable to assess. In addition, while hospital readmission decreased at one year, we are unable to further differentiate reasons associated with the return to the hospital. Further study is necessary to determine and characterize the potential causality of these relationships.

Nevertheless, optimizing post-operative pain control and decreasing healthcare utilization after arthroscopic RCR remain important as the rate of RCR increases. The findings of this study suggest that perioperative TXA with RCR decreases the number of post-operative opioid prescriptions and coded healthcare utilization. While further prospective study is needed to clarify the mechanisms underlying these associations and determine dose- and duration-dependent effects, the study findings support that the benefits of perioperative TXA extend beyond the immediate post-operative period after arthroscopic RCR.

## Conclusions

In this large, propensity-matched database study, perioperative use of TXA with arthroscopic RCR was associated with fewer post-operative opioid prescriptions at 30 days and one year post-operatively and lower coded rates of healthcare utilization at one year, but not 30 days, without an increase in recorded thromboembolic complications. Given the associative nature of these findings, they should be considered hypothesis-generating and warrant further prospective validation.

## Disclaimers:

Funding: No funding was disclosed by the authors.

Conflicts of interest: The authors, their immediate families, and any research foundations with which they are affiliated have not received any financial payments or other benefits from any commercial entity related to the subject of this article.
