# Supplementary material for: Perioperative outcomes following tranexamic acid use in arthroscopic rotator cuff repair
Source: JSES Int. 2026 Jul 27;10(5):101769. doi: 10.1016/j.jseint.2026.101769 (PMC13524671; doi:10.1016/j.jseint.2026.101769)
Supplement: Supplementary file 1 [file mmc1.docx]

Supplemental Table 1: International Classification of Diseases, Current Procedural Terminology, Anatomic Therapeutic Chemical Class, and Veteran Affairs Formulary codes used to assess postoperative outcomes.

| Outcome | ICD/CPT/SNOMED codes |
| --- | --- |
| Readmission | CPT 1013699, SNOMED 737481003, 394656005, 32485007, 50699000, 76464004, 183461005, 74857007, 305349003, 281685003, 304566005, 305260005, 305293001, 305224000 |
| Emergency Department Visit | CPT 99281, 99282, 99283, 99284, 99285, 1013711, 1013723, SNOMED 385868005, 50849002 |
| Superficial Surgical Site Infection | ICD-10-CM T81.4, L76.32, M96.840, M96.841, M96.842, M96.843, L76.34, T81.30XA, T81.31XA, T81.4XXA, L03.019, L03.113, L03.114, L08.9 |
| Pulmonary Embolism | ICD-10-CM I26, I26.93, I26.99, I26.09, I26.92, I26.02 |
| Deep Vein Thrombosis | ICD-10-CM I82.40, I82.409, I82.890, T81.719A, I82.419, I82.429, I82.439, I82.449 |
| Acute Renal Failure | ICD-10-CM N17, N99.89, N28, N99.0, N17.0, N17.1, N17.2, N19 |
| Aspiration | ICD-10-CM J69.0, J69.8, J95.89, J95.859, J95.4 |
| Mortality | ICD-10-CM R99, G93.82, SNOMED 41819004, 307928008, 184274007 |
| Sepsis | ICD-10-CM A40, A41 |
| Urinary Tract Infection | ICD-10-CM N39 |
| Deep Surgical Site Infection | CPT 29822, 29823, 23040, 23031,23030 |
| Opioid prescriptions | VA CN 101, ATC N01AH |
